# Case Report: An incidental finding of an metastases noted in a “cancer to cancer adrenal tumor ” from a large malignant nerve sheath tumor of the thigh

**DOI:** 10.12688/f1000research.12647.2

**Published:** 2018-04-09

**Authors:** Matthew D. Shaines, Shitij Arora

**Affiliations:** 1 Department of Internal Medicine, Division of Hospital Medicine, Montefiore Medical Center, Albert Einstein College of Medicine, Bronx, New York City, NY, USA

**Keywords:** nerve sheath tumor, adrenal, lung, metastasis

## Abstract

Current guidelines are vague for the management of soft tissue sarcomas, specifically malignant peripheral nerve sheath tumors (MPNST), regarding staging the disease with the use of routine abdominal imaging. The most recent guidelines from the National Comprehensive Cancer Network (NCCN) recommends to “consider” abdominal/pelvic CT imaging for certain sub groups of sarcomas (e.g., myxoid/round cell liposarcoma, epithelial sarcoma, angiosarcoma,  leiomyosarcoma), but provide no guidance on other sarcoma subtypes regardless of tumor size. We report a case of a very large large MPNST  in a 40 year-old-female with neurofibromatosis type 1 who was incidentally found to have adrenal metastasis.

## Introduction

Soft tissue sarcomas of the extremity are a rare disease process, comprising less than 1% of all malignancies ^1^. The majority of soft-tissue sarcomas occur in the limb or limb girdle or within the abdomen, with 40% being found in the lower extremities. These sarcomas are a histologically heterogeneous group of tumors (over 50 tumor subtypes have been identified) with a predilection for hematogenous spread. Distant metastatic disease is found in approximately 20–30% of patients ^1– 3^, with pulmonary lesions accounting for 75% of these cases ^1, 4, 5^. Because of the relatively low incidence of extra-pulmonary metastasis, the current guidelines from the National Comprehensive Cancer Network (NCCN) recommends to only “consider” abdominal/pelvic CT imaging for certain subgroups of sarcomas (myxoid/round cell liposarcoma, epithelial sarcoma, angiosarcoma, leiomyosarcoma) ^6^. A similar recommendation is made by the European Society of Medical Oncology (ESMO)/European Sarcoma Network Working Group ^7^. No specific recommendation is made for any additional sarcoma subtype regardless of size of the tumor, despite this being a known prognostic factor.

Malignant peripheral nerve sheath tumors (MPNST) are highly malignant sarcomas which originate from peripheral nerves or from cells associated with the nerve sheath, such as Schwann cells, perineural cells, or fibroblasts. MPNSTs compromise 5–10% of all soft tissue sarcomas with 22–50% of the tumors associated with a diagnosis of Neurofibromatosis-1 (NF1) ^8– 10^. We report a case of a malignant peripheral nerve sheath tumor of the thigh, without evidence of concomitant pulmonary metastases, which was found to have metastasized to the adrenal gland. Thus, we pose the question of should the staging work up for large sarcomas be expanded to include abdominal imaging, even if the CT chest is unrevealing?

## Case report

**Figure 1.**
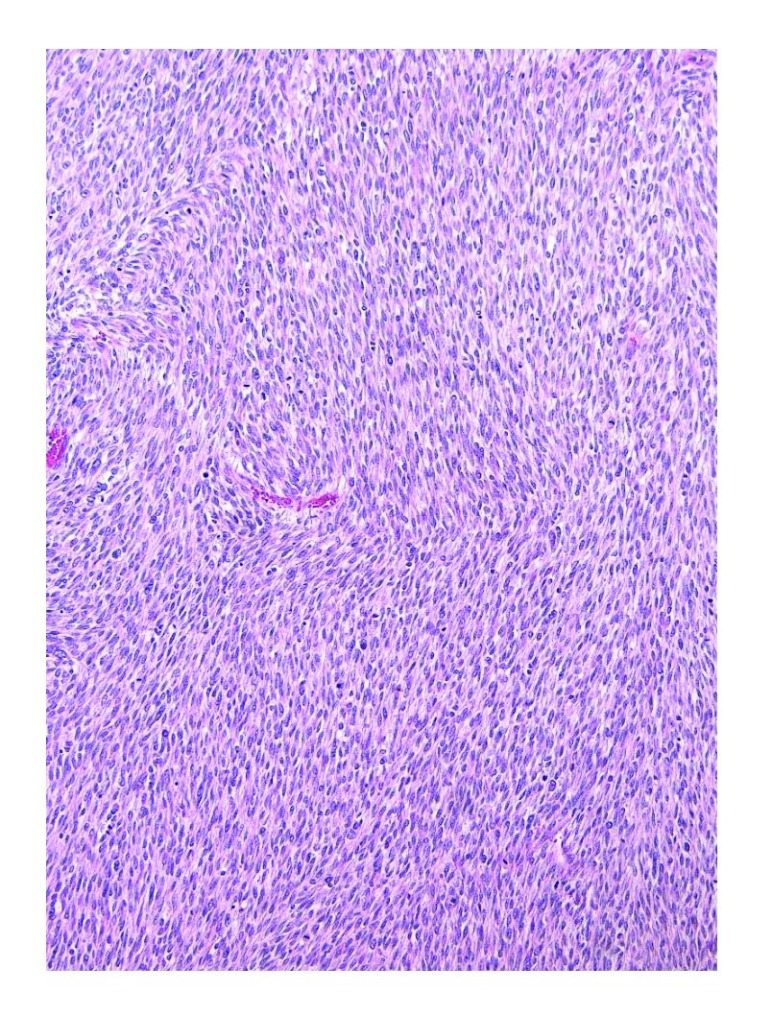
Section from the thigh mass (10x, H&E) shows a hypercellular tumor, with spindle cells in sheets and fascicular arrangement. The spindle-shaped nuclei have clumped chromatin. These features are compatible with a malignant peripheral nerve sheath tumor.

**Figure 2.**
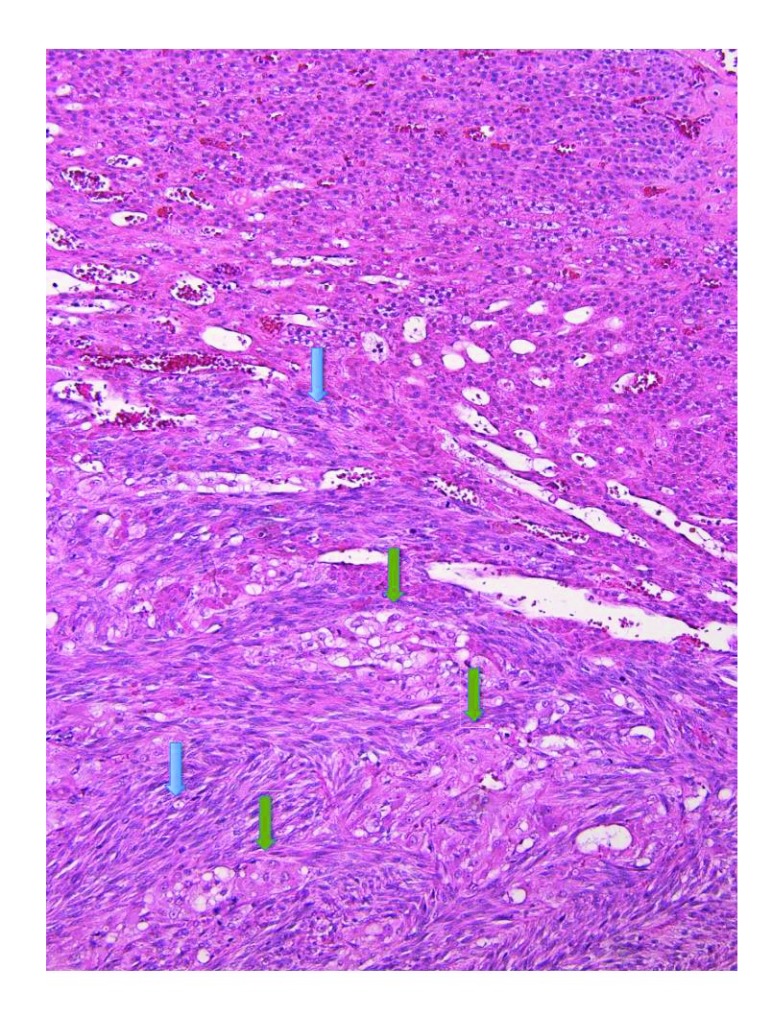
The area on the right shows two populations of tumor cells that are intermingling with each other, representing a collision tumor (20x, H&E). One population is composed of hypercellular malignant spindle cells with hyperchromatic nuclei (blue arrow) that are infiltrating the adjacent adrenal tissue. This is morphologically compatible with malignant peripheral nerve sheath tumor. The other population is composed of the nests of polygonal cells with abundant eosinophilic cytoplasm (green arrow), compatible with pheochromocytoma.

A 40 year old woman withNF1 presented for a hemorrhagic right thigh mass which had been enlarging over the past 3 months. The mass had previously been stable in size for a few years and was thought to be consistent with a neurofibroma. On presentation her exam was notable for a very large soft tissue tumor of the right posterior upper leg (>15cm). The distal portion of the lesion revealed skin breakdown and active bleeding with exposed muscle and tumor. Laboratory analyses revealed a white blood cell count of 23.0k/µL and hemoglobin of 10.7 g/dL with an unremarkable basic metabolic panel. Computerized tomogram (CT) of the extremities revealed a large heterogeneously enhancing mass within the posterior compartment of the right thigh, measuring 13.4 × 13.4 × 24.8 cm. In addition, an incidental 2 × 2 cm left adrenal gland mass was noted. Of note, a CT scan of the thorax, completed for staging purposes, did not reveal any suspicious pulmonary nodules or masses. After an initial excisional biopsy of the thigh mass, she underwent a radical resection of the right thigh mass. Pathology confirmed a high grade spindle cell sarcoma, negative for S100 protein and SOX10 (variable expression in MPNST), Desmin (rhabdomyosarcoma differentiation), CD31 and AE1:AE3 (vascular sarcomas, myoepitheliomas), HMB45, MelanA (epitheloid MPNST). Given the clinical history of NF-1 and the lack of immunoreactivity for all performed markers for this tumor was most compatible with an undifferentiated MPNST ( Figure 1) AMRI of the abdomen, to further evaluate the adrenal mass, revealed 2 small left adrenal gland lesions in the medial and lateral limbs. Given the known association of neurofibromatosis with pheochromocytoma, a biochemical workup was pursued and confirmed this diagnosis. The patient underwent a laparoscopic adrenalectomy, with gross pathological exams revealing two tumor nodules and with a histological exam revealing an intermixed “cancer to cancer metastasis” involving pheochromocytoma and sarcoma, consistent with MPNST( Figure 2). The patient refused radiation therapy and did not follow up with oncology.

## Discussion

The above case describes a patient with a MPNST of the thigh with pathologically confirmed metastases to the adrenal gland, yet without evidence of pulmonary metastases. The adrenal mass was found incidentally upon imaging of the thigh mass, but based on current NCCN guidelines ^6^, a screening CT of the abdomen/pelvis (A/P) would not be indicated and thus could have potentially missed the presence of metastatic disease.

To the best of our knowledge there have only been 2 case series that comment on the indication for A/P imaging in sarcoma patient and they reached contradictory conclusions.. In the first case series, King, *et al*. evaluated 124 adult patients with sarcoma who underwent CT chest(C)/A/P imaging at their institution for staging and surveillance. Twenty (16%) of the patients had evidence of A/P metastasis, 7 on the initial scan and 13 on the surveillance ^11^. Of note, six of the 20 patients (5% of the cohort) were found to have isolated A/P metastases without the development of pulmonary metastases during the study period. MPNST, specifically, made up 6% of the sarcomas evaluated and while no A/P metastases were found on screening, 2 patients had evidence on surveillance scans. Based on the finding that a wide variety of sarcoma subtypes were found to have extra-pulmonary disease, the authors conclude that A/P imaging should be included in the evaluation of all sarcoma subtypes. In contrast, Thompson *et al*. reviewed 140 patients of all ages who had a diagnosis of a malignant neoplasm of the upper or lower extremity and underwent screening and/or surveillance with a CT C/A/P ^12^. Of those patients, 14 (10%) had evidence of abdominal/pelvic metastasis, with only 4 (2.9%) with evidence of isolated A/P disease. Additionally, of the 10 patients who developed metastases to both the chest and abdomen/pelvis, none developed evidence of disease in the abdomen/pelvis prior to the chest. Of note, though, there were only 2 MPNSTs in the entire cohort and neither one had evidence of abdominopelvic disease on imaging. Based on their results, the authors offer up the contrary opinion from King and do not support routine abdomen/pelvis imaging.

Our case adds a patient to the literature with a peripheral MPNST who was found to have an isolated adrenal metastasis without evidence of concomitant pulmonary disease. One striking feature of this case is the very large size of the primary tumor(>20 cm). Factors known to be associated with the development of metastases are tumor grade, tumor size, tumor depth, and certain histopathologies. Specifically, tumors > 5cm have been found to be associated with an increased risk of metastatic recurrence ^13^. Currently, though, guidelines only recommend CT of the chest for all patients and to consider CT A/P in patients with myxoid/round cell liposarcoma, epithelial sarcoma, angiosarcoma and leiomyosarcoma. Based on our case and the literature, we also would suggest adding screening CT A/P for large (>5 cm), deep tumors of any histology.

## Conclusions

Soft tissue sarcomas are a heterogeneous group of tumors with a variety of prognoses. Deciding on a unified set of guidelines will be challenging, but given the clinical significance of finding metastatic disease, adding additional parameters (size and depth) to a more complete screening process would seem prudent.

## Consent

Written informed consent was obtained by the patient for publication of their clinical details. There are no potentially identifying images included in this paper.
