# Supplementary material for: Case Report: An incidental finding of an metastases noted in a “cancer to cancer adrenal tumor ” from a large malignant nerve sheath tumor of the thigh
Source: F1000Res. 2018 Apr 9;6:1964. Originally published 2017 Nov 7. [Version 2] doi: 10.12688/f1000research.12647.2 (PMC5954341; doi:10.12688/f1000research.12647.2)
Supplement: Peer review history

# Peer review: Referee response for version 2

**DOI:** 10.5256/f1000research.15689.r32955

**Published:** 2018-05-14

**Authors:** Shweta Gera

**Affiliations:** 1 Department of Pathology, Montefiore Medical Center, Bronx, NY, USA

Authors have addressed the majority of the concerns except few. Eg. No correction of epithelial sarcoma to epithelioid sarcoma, expanding on the physical and gross examination for thigh mass and adrenal metastasis. There is some problem with spacing between the words as well, eg- "AMRI", "withNF1". Please consider addressing these issues.

I have read this submission. I believe that I have an appropriate level of expertise to confirm that it is of an acceptable scientific standard.

# Peer review: Referee response for version 2

**DOI:** 10.5256/f1000research.15689.r32956

**Published:** 2018-04-13

**Authors:** Ahmed Abu-zaid

**Affiliations:** 1 College of Graduate Health Sciences, University of Tennessee Health Science Center, Memphis, TN, USA

With interest, I read the revised version of manuscript. Authors have addressed almost all the comments. The manuscript needs minor English polishing prior to final publication (for example, the title mentions "an metastases") and many others throughout the manuscript.

I have read this submission. I believe that I have an appropriate level of expertise to confirm that it is of an acceptable scientific standard.

# Peer review: Referee response for version 1

**DOI:** 10.5256/f1000research.13693.r29117

**Published:** 2018-01-08

**Authors:** Shweta Gera

**Affiliations:** 1 Department of Pathology, Montefiore Medical Center, Bronx, NY, USA

The case report describes an interesting finding but it needs major revision. Some of the changes suggested as below-

  

- It describes an NF type-1 patient with MPNST with metastasis to the adrenal gland. The patients with NF type-1 have increased risk for pheochromocytoma. Likely the patient had undiagnosed pheochromocytoma (was the patient hypertensive?) and the patient developed metastasis to pheochromocytoma. So it describes more of cancer-to-cancer metastasis than collision tumor. Add more references with respect to cancer-to-cancer metastasis. Collision tumor is defined as two distinct tumors developing in juxtaposition to one another without areas of intermingling. In the current case, as in Figure 2, there is an intermingling of both components- so again it is not a collision tumor. The title needs to be changed and make it apt.
- Spelling errors like prognoses (mentioned in conclusion). There is nothing called epithelial sarcoma, the correct terminology is epithelioid sarcoma. MPNST stands for malignant peripheral nerve sheath tumor so no need for peripheral MPNST (mentioned in the first line of the last paragraph of discussion). Please check for grammatical errors as well.  
- Abstract- talks about guidelines for staging peripheral nerve sheath tumors, which sounds like it was a benign tumor. So use MPNST instead of just peripheral nerve sheath tumor. Abstract and discussion mention isolated adrenal metastasis, which is not true; she had both bone and adrenal metastasis. There is mention that- "She underwent primary and adrenal metastasis resection." consider revising it to primary tumor resection and adrenalectomy for metastasis. 
- Expand on physical exam of thigh mass- size, tenderness, consistency etc. Elaborate on findings of MRI with details of imaging findings of adrenal metastasis.  
- Expand on gross findings of thigh mass and adrenal nodules- eg. size, color, infiltrative or well-circumscribed etc. Did both the adrenal nodules have similar histological findings? Were any immunostains done to confirm pheochromocytoma component?  
- Ref 9 talks about patients with malignant neoplasm of the upper or lower extremity. There is no mention of that those were sarcomas. Check for that.  
- Expand on abstract- add more about the significance of the findings of the case; expand on introduction and discussion- add more details for sarcoma, development of MPNST in association to NF-1, adrenal metastasis, management guidelines, more references for the recommendation for screening CT A/P for sarcomas. In the discussion, authors have suggested adding screening CT A/P for large (>5cm), deep tumors of any histology- expand more on this by adding more references.  
- Add limitations of the study, the major limitation being small sample size.

I have read this submission. I believe that I have an appropriate level of expertise to confirm that it is of an acceptable scientific standard, however I have significant reservations, as outlined above.

# Response

**Published:** 2018-03-23

**Authors:** Shitij Arora

**Affiliations:** Montefiore Medical Center, Albert Einstein College of Medicine, USA

We thank you for the review and the excellent suggestions. Our response is included below

t describes an NF type-1 patient with MPNST with metastasis to the adrenal gland. The patients with NF type-1 have increased risk for pheochromocytoma. Likely the patient had undiagnosed pheochromocytoma (was the patient hypertensive?) and the patient developed metastasis to pheochromocytoma. So it describes more of cancer-to-cancer metastasis than collision tumor. Add more references with respect to cancer-to-cancer metastasis. Collision tumor is defined as two distinct tumors developing in juxtaposition to one another without areas of intermingling. In the current case, as in Figure 2, there is an intermingling of both components- so again it is not a collision tumor. The title needs to be changed and make it apt.

Response  - term collision tumor is removed.

Spelling errors like prognoses (mentioned in conclusion). There is nothing called epithelial sarcoma, the correct terminology is epithelioid sarcoma. MPNST stands for malignant peripheral nerve sheath tumor so no need for peripheral MPNST (mentioned in the first line of the last paragraph of discussion). Please check for grammatical errors as well.  

 Response - corrected

Abstract- talks about guidelines for staging peripheral nerve sheath tumors, which sounds like it was a benign tumor. So use MPNST instead of just peripheral nerve sheath tumor. Abstract and discussion mention isolated adrenal metastasis, which is not true; she had both bone and adrenal metastasis. There is mention that- "She underwent primary and adrenal metastasis resection." consider revising it to primary tumor resection and adrenalectomy for metastasis.

 Response - corrected

Expand on physical exam of thigh mass- size, tenderness, consistency etc. Elaborate on findings of MRI with details of imaging findings of adrenal metastasis.  

 Response- corrected

Expand on gross findings of thigh mass and adrenal nodules- eg. size, color, infiltrative or well-circumscribed etc. Did both the adrenal nodules have similar histological findings? Were any immunostains done to confirm pheochromocytoma component?  

Response- immunostains are included in the findings described

dd limitations of the study, the major limitation being small sample size.

Response- We intend to publish this as a case report

# Peer review: Referee response for version 1

**DOI:** 10.5256/f1000research.13693.r29129

**Published:** 2017-12-20

**Authors:** Ahmed Abu-zaid

**Affiliations:** 1 College of Graduate Health Sciences, University of Tennessee Health Science Center, Memphis, TN, USA

With interest, I read the article Shaines and Arora. Overall, the article reads very well, and should deserve acceptance following some minor/major changes to improve its quality and scientific soundness.

 1. Title:

 — You may want remove the phrase "case report".

 — Also, please change to "an adrenal metastasis".

 2. Abstract.

 — Please expand the abstract.

 — You may want to include a couple of sentences about definition of soft tissue sarcomas (STSs), incidence, biological behavior (with respect to abdominal/pelvis and distant metastasis) and percentage of MPNSTs.

 — You may also want to precisely mention the names of guidelines and dates.

 — All these recommended sentences above will highlight the importance of your case report.

 — The abstract says the STS of thigh was resected although this is not mentioned in the "case report" section. 

 — Lastly, check if journal mandates a STRUCTURED format for the abstract section. 

 3. Introduction:

 — It is recommended to add sentences that shed light on the most common sites of STSs and the percentage of MPNSTs among all STSs.

 — Mention the dates of guidelines.

 4. Case Report:

 — Add details about physical examination.

 — Add details about initial laboratory findings.

 — Add CT scans of the thigh mass (to highlight its large size) and adrenal mass (to highlight the collision tumor).

 — You add the MRI picture of the adrenal mass, too (or the CT scan).

 — Please do mention of the patient received surgery for the STS of the thigh as it is not clearly mentioned in this section.

 5.  Discussion:

 — Since the two series presented in manuscript had contradictory conclusions, it is recommended (if available) to add additional data from one to two more series. 

 — For reference (9), please add specific details about MPNSTs (if available).

 — Briefly mention how (why) MPNST are different from the myxoid liposarcoma, epithelial sarcoma, angiosarcoma etc in terms of the recommendation for CT A/P scanning.

 — please provide citations for the following sentence "Factors known to be associated with the development of metastases are tumor grade, tumor size, tumor depth, and certain histopathologies".

 5. "Based on our case and the literature, we also would suggest adding screening CT A/P for large (>5cm), deep tumors of any histology". How depth was assessed in your case report.

 6. Conclusion:

 — Modify the conclusion so it will reflect the specific histopathological variant of MPNST (since it is not normally recommended by guidelines as opposed to the others).

 7. References:

 — Good

 8. English:

 — Minor English polishing.

I have read this submission. I believe that I have an appropriate level of expertise to confirm that it is of an acceptable scientific standard, however I have significant reservations, as outlined above.

# Response

**Published:** 2018-03-23

**Authors:** Shitij Arora

**Affiliations:** Montefiore Medical Center, Albert Einstein College of Medicine, USA

We thank the reviewer for their excellent suggestions. Our response is included below

Reviewers Response /1

Abstract. — Please expand the abstract. — You may want to include a couple of sentences about definition of soft tissue sarcomas (STSs), incidence, biological behavior (with respect to abdominal/pelvis and distant metastasis) and percentage of MPNSTs. — You may also want to precisely mention the names of guidelines and dates. — All these recommended sentences above will highlight the importance of your case report. — The abstract says the STS of thigh was resected although this is not mentioned in the "case report" section.  — Lastly, check if journal mandates a STRUCTURED format for the abstract section.

Response- We  included much of the above in an updated version of the introduction

Introduction: — It is recommended to add sentences that shed light on the most common sites of STSs and the percentage of MPNSTs among all STSs. — Mention the dates of guidelines.

Response -Updated

Case Report: — Add details about physical examination. — Add details about initial laboratory findings. — Add CT scans of the thigh mass (to highlight its large size) and adrenal mass (to highlight the collision tumor). — You add the MRI picture of the adrenal mass, too (or the CT scan). — Please do mention of the patient received surgery for the STS of the thigh as it is not clearly mentioned in this section.

Response- Updated much of this.

We have attached an image of the thigh mass with this response instead – please click here to see this.

Discussion: — Since the two series presented in manuscript had contradictory conclusions, it is recommended (if available) to add additional data from one to two more series.  — For reference (9), please add specific details about MPNSTs (if available). — Briefly mention how (why) MPNST are different from the myxoid liposarcoma, epithelial sarcoma, angiosarcoma etc in terms of the recommendation for CT A/P scanning. — please provide citations for the following sentence "Factors known to be associated with the development of metastases are tumor grade, tumor size, tumor depth, and certain histopathologies". 5. "Based on our case and the literature, we also would suggest adding screening CT A/P for large (>5cm), deep tumors of any histology". How depth was assessed in your case report.

Response -Updated much of this

These are the only 2 case series we could find that address this issue of A/P imaging

we removed “depth” from that last sentence as it really does appear that size if what matters

Conclusion: — Modify the conclusion so it will reflect the specific histopathological variant of MPNST (since it is not normally recommended by guidelines as opposed to the others).

Response- Wepurposely left this vague because this this was only one case of MPNST and there are so many variants of soft tissue sarcoma. I thought it would be more generalizable to state tumor size, in general, should be thought of as important when it comes to screening imaging since it is noted as a risk factor for all STS.
